# Prevalence and antimicrobial susceptibility of asymptomatic bacteriuria among women with pelvic organ prolapse in Abakaliki, South-East Nigeria

**DOI:** 10.1186/s12905-018-0545-9

**Published:** 2018-03-27

**Authors:** K. C. Ekwedigwe, I. Sunday-Adeoye, M. O. Eliboh, M. E. Isikhuemen, H. Uro-Chukwu, P. Ezeonu, A. B. C. Daniyan, E. N. Yakubu

**Affiliations:** 1 National Obstetric Fistula Centre, Abakaliki, Nigeria; 2 Federal Teaching Hospital, Abakaliki, Nigeria

**Keywords:** Pelvic organ prolapse, Asymptomatic bacteriuria, Abakaliki, Nigeria

## Abstract

**Background:**

Pelvic organ prolapse (POP) is the herniation of pelvic organs from its anatomical confines, and it is of considerable importance to the practicing gynaecologist in middle and low income countries. It is commonly associated with, urinary tract infection (UTI), both symptomatic and asymptomatic due to anatomical and physiological changes. The aim of this study was to determine the prevalence of asymptomatic bacteriuria among women with pelvic organ prolapse, to know the organisms commonly implicated and the sensitivity pattern.

**Methods:**

This study was conducted among 96 women with POP at the National Obstetric Fistula Centre Abakaliki. A cross sectional descriptive study was done. Standard microbial technique was used to analyze the urine. Data was analysed using the Statistical Package for Social Sciences version 17.

**Results:**

Out of the 96 patients, 76 were found to have asymptomatic bacteriuria giving a prevalence of 79.2%. Nine different bacteria species isolated include E. Coli (34.2%), Streptococcus pneumonia (23.7%), Staphylococcus aureus (7.9%), Proteus Spp (7.9%) others (5.3%). The highest level of microbial sensitivity to the antimicrobials was with Ciprofloxacin.

**Conclusions:**

This study suggests that prevalence of asymptomatic bacteriuria is very high among women with POP. More than 50% of the bacterial isolates were mainly E.coli and Streptococcus pneumonia. The highest level of microbial sensitivity was with ciprofloxacin while the least was with cotrimoxazole.

## Background

Pelvic organ prolapse (POP) is the herniation of the pelvic organs from its anatomical confines [1]. It is a common gynaecological problem particularly in the grandmultipara [1–8]. It is of considerable importance to the practicing gynaecologist in the developing countries because of its strong association with repeated child bearing [2–4, 8–10]. Poor conduct of labour can predispose to uterine prolapse [3, 11, 12].

Pelvic organ prolapse is also common in conditions of chronically raised intra-abdominal pressure [2–4, 11]. Very rarely, it could be due to congenital weakness of the pelvic floor muscles and altered collagen metabolism [3, 12].

The prevalence of asymptomatic bacteriuria is 1.0%–5.0% in healthy population [13]. Pelvic organ prolapse (POP) is a very common problem with a prevalence of 41–50% in women over the age of 40 years. It was reported as present in 50% of parous women, with the aggregated rates of prolapse surgery which were estimated at between 15 and 49 per 10,000 per women year [14, 15].

It is commonly associated with, urinary tract infection, UTI (both symptomatic and asymptomatic) due to anatomical and physiological changes [1]. Asymptomatic bacteriuria is the presence of live bacteria in the urine of an individual without symptoms of UTI [10]. Significant bacteriuria is the finding of 100,000 pure colonies in 1 ml of uncentrifuged urine sample [11]. Further, with the abuse of antibiotics, resistant bacterial strains are likely to emerge as major microbial isolates in urine, thus further complicating the management [12].

Escherichia coli remains the single most common organism isolated from bacteriuric women [13, 16, 17] Other Enterbacteriaceae (such as Klebsiella pneumonia) and other organisms (including coagulase negative staphylococci, Enterococcus species, group B streptococci, and Gardnerella vaginalis) are common as well [13].

Prolapse classically produces a sensation of fullness in the vagina or a visible or palpable lump at the introitus. While women who have prolapse may have stress incontinence, particularly if the urethra is not well supported, they may also have voiding dysfunction secondary to kinking for the urethra [1]. Voiding dysfunction may result in frequency (due to incomplete bladder emptying), hesitancy and a poor urinary stream. This results in stasis of urine in the urinary tract, which is culture medium for bacteria growth [1]. Incomplete bladder emptying may in turn result in recurrent urinary tract infection with accompanying frequency, urgency and urge incontinence. Most cases of asymptomatic bacteriuria are subclinical UTI. This can be exacerbated by surgery and other stressors [1, 4, 5].

This aspect of knowledge is so sparsely studied and there is paucity of literature bank in this area. Our aim was to determine the prevalence and antimicrobial susceptibility of asymptomatic bacteriuria among women with pelvic organ prolapse.

## Methods

The study was conducted at the National Obstetric Fistula Centre, Abakaliki which offers free surgical services to patients with urogenital fistula and pelvic organ prolapse. A cross sectional descriptive study was done. Study population was women with pelvic organ prolapse presenting at the centre for surgery. Patients with pelvic organ prolapse without symptoms of urinary tract infection were included in this study. Women with symptoms of UTI and those who refused to give consent were excluded. The study was carried over a 12-month period (Ist July 2013 – 30th June 2014).

The sample size was calculated by a statistical formula based on the proportion of 3.91% [18] for women with POP from a study by Ojiyi et al. in Imo State, South East Nigeria, and a confidence level set at 95% with an error margin of 0.05.

Following informed consent, a questionnaire was used to obtain relevant information from the study participants. Midstream urine was collected from each of the subjects using a urethral catheter into sterile containers (universal bottles). Urine samples were cultured on cystine lactose electrolyte deficiency (CLED) and blood agar (BA) medium [19, 20]. The culture plates were incubated at 37 °C for a duration of 24 h. Several biochemical tests were conducted for bacterial isolation and identification. Bacteria isolates made were subjected to antibiotic sensitivity analysis using the disc diffusion methods [20, 21]. The sensitive drugs were gentamycin, ciprofloxacin, ceftriaxone, ofloxacin, perfloxacin, cotrimoxazole, cefuroxime Tetracycline, Chloramphenicol, Cloxacillin, Streptomycin, Nalidixic acid, Augmentin, Erythromycin, Amoxicillin and Nitrofurantoin. Study participants with significant bacteriuria were treated accordingly.

Collected data was analyzed using the Statistical Package for Social Sciences (SPSS) version 17.

Ethical approval was obtained from the Ethics and Research committee of the National Obstetric Fistula Centre, Abakaliki. Individual informed consent form was attached to each questionnaire and the respondent gave her consent before the questionnaires were filled.

## Results

The findings of this study are presented as follows:

The age groups 40–49 years and 50–59 years constituted the greatest percentage with 25.0% and 33.3% respectively. This is as shown in Table 1.

**Table 1:** Social demographic variables

Variables	Frequency (%)
Age	
20–29	2 (2.1)
30–39	13(13.5)
40–49	24(25.0)
50–59	32(33.3)
60–69	20(20.8)
70–79	3(3.1)
> 79	2(2.1)
Median age = 51	
Parity	
2–4	16(6.6)
5–7	51(53)
8–10	25(26)
> 10	4(4.2)
Median parity = 6	
Marital Status	
Married	75(78.1)
Divorced	6(6.3)
Widow	15(15.6)
Level of Education	
No formal education	81(84.4)
Part Prim. Education	9(9.4)
Primary education	6(6.2)
Occupation	
Unemployed	23(24.0)
Unskilled	65(67.7)
Middle level	8(8.3)
Husband Occupation	
Unemployed	20(20.8)
Unskilled	51(53.1)
Middle level	11(11.5)
Professional	14(14.6)

Out of the 96 women with pelvic organ prolapse recruited, 76 of them were positive for asymptomatic bacteriuria. This makes the prevalence of asymptomatic bacteriuria to be 79.2%.

For clients without asymptomatic bacteriuria, mean parity was 6.75 ± 2.83.

Nine (9) different bacteria species were isolated (Table 2) More than half of the bacteria isolated from the urine of the patients were E. coli (34.2%) and Streptococcus pneumonia (23.7%).

**Table 2:** Bacteria isolated from urine of Participants (*n* = 76)

Common Organism implicated	Frequency (%)
Strept faecalis	4 (5.3)
Staph aureus	6(7.9)
Strept pneumonia	18(23.7)
Proteus spp	6(7.9)
E. coli	26(34.2)
Klepsiella spp	4(5.3)
Strept pyogenes	4(5.3)
Pseudomonas aeruginosa	4(5.3)
Enterobacter	4(5.3)
Total	76(100)

The highest level of microbial sensitivity to the antimicrobials was with ciprofloxacin while the least was with cotrimoxazole (Table 3).

**Table 3:** Sensitivity/Resistance pattern of various organisms isolated from urine of Patients

Organism	Sensitivity	Resistance
Strept faecalis	Tetracycline, Chloramphenicol, Cloxacillin, Streptomycin, Augmentin, Gentamycin, Ciprofloxacin	Erythromycin, Cotrimoxazole
Staph aerueus	Gentamycin, Streptomycin, Augmentin, Chloramphenicol, Cloxacillin	Erythromycin, Cotrimoxazole, Ciprofloxacin, Tetracycline
Strept pneumonia	Augmentin, Cloxacillin, Chloramphenicol, Ciprofloxacin	Gentamycin, Erythromycin, Cotrimoxazole, Tetracycline, Streptomycin
Proteus spp	Nitrofurantoin, Ciprofloxacin	Cotrimoxazole, Tetracycline, Augmentin, Gentamycin, Nalidixic acid, Amoxicillin, Ofloxacin
Escherichia coli	Nitrofurantoin, Ofloxacin, Gentamycin, Nalidixic acid, Ciprofloxacin	Cotrimoxazole, Amoxicillin, Tetracycline, Augmentin
Klebsiella spp	Streptomycin, Cotrimoxazole, Gentamycin, Augmentin, Chloramphenicol, Ciprofloxacin	Tetracycline, Cloxacillin, Erythromycin
Strept pyogenes	Chloramphenicol, Augmentin, Ciprofloxacin, Cloxacillin	Gentamycin, Streptomycin, Tetracycline, Cotrimoxazole
Pseudomonas aeruginosa	Gentamycin	Nalidixic acid, Cotrimoxazole, Amoxicillin, Tetracycline, Augmentin, Ofloxacin, Nitrofurantoin
Enterobacter	Ofloxacin, Gentamycin, Nalidixic acid, Nitrofurantoin	Cotrimoxazole, Amoxicillin, Tetracycline, Augmentin

## Discussion

The age group 40–49 years and 50–59 years constituted the greatest percentage of the patients (25%) and (33.3%) respectively with POP and the mean age was 52.79 years with standard deviation of 10.84 years. These sociodemographic variables are similar to studies done in Lagos [2], Enugu [3], India [4], Ibadan [9], Nnewi [12]. This could be due to reduction in the production of female hormones especially estrogen during the perimenopausal and menopausal age range and the subsequent effect of these reduced hormones in the pelvic supporting structures [22] .Secondly, most women in this age range are multipara with its attendant effect on the ligaments and other pelvic supporting organs [2, 18, 21]. Majority of the women were grandmultiparous (that is 5 or more deliveries) with highest value being Para 6(18.8%).The mean Parity was 6.74 with standard deviation of 2.27 and the median parity was 6. This is also similar to studies done in Lagos [2], Enugu [3], India [4], Ibadan [9], Nnewi [12], Benin [23], where the majority of the women were grandmultiparous and the mean parities were 6 and above. Pregnancy and bearing down in the second stage of labour further puts much more tension to these pelvic supporting structures thereby predisposing these women to developing pelvic organ prolapse [1]. Prolonged labour that is labour lasting more than 12 h and unsupervised deliveries are strong determinants [21]. Increased number of pregnancies will cause recurrent repetition of these events, thus, the higher the number of pregnancies, the higher the risk of developing pelvic organ prolapse [1, 8].

The results of this study showed that the prevalence of asymptomatic bacteriuria was 79.2% among women with pelvic organ prolapse. This is greater than the figures from study done in Ekiti state, Nigeria which found a prevalence of 10% among antenatal enrollees in southwest Nigeria [24]. The prevalence is generally far less in pregnant women (< 20%) [24–28]. This could be due to increase kinking of ureters, hydroureter, hydronephrosis and stasis of urine in POP patients compared with normal pregnant women. This is greater than the figures from studies done in Mexico (61%) [29]. This could be because of improved personal hygiene and differences in life style among the study population, more so, the Mexican study was a multicenter study. It is also low in a community based study among elderly population done in a Swedish mid-sized town, where the prevalence was 14.8% [22]. This could be because the elderly people used in this study were apparently healthy population and because of improved personal hygiene and life style differences.

In this study, nine (9) bacteria species were isolated from the patients and they included Escherichia coli (34.2%), Streptococcus pneumonia (23.7%), Staphylococcus aureus (7.9%), Proteus species (7.9%), Klebsiella species (5.3%), Streptococcus pyogenes (5.3%), Pseudomonas aeruginosa (5.3%) and Enterobacter species (5.3%). The commonest organisms implicated in asymptomatic bacteriuria were E. coli (34.2%), and Streptococcus pneumonia (23.7%). In the community based study on asymptomatic bacteriuria among the elderly population done in Sweden, E.coli was the predominant organism implicated likewise the study on asymptomatic bacteriuria among the vesico-vaginal fistula clients done in South-East Nigeria and the study on asymptomatic bacteriuria in children done in Sweden, E. coli was named as the commonest organism [24, 29–33]. Most studies on asymptomatic bacteriuria in pregnancy also implicated E.coli as the predominant organism [27, 28]. This could be explained due to the fact that it is a normal flora in the lower gastrointestinal tract, and also due to its adhesive ability.

The highest level of microbial sensitivity to the antimicrobials was with ciprofloxacin while the least was with cotrimoxazole. Strept.faecalis was resistant to only erythromycin and cotrimoxazole.

This study is limited by its sample size. Also, a probability sampling method was not used. This should form the basis for future research.

## Conclusions

The prevalence of asymptomatic bacteriuria in women with POP presenting for surgery at the National Obstetric Fistula Centre from this study was rather very high (79.2%). Several microorganism (E. Coli, Streptococcus pneumonia, Staphylococcus aureus, Proteus Spp, Strept faecalis, Klebsiella spp, Enterobacter, Strept pyogenese, Pseudomonas aeruginosa) were implicated in asymptomatic bacteriuria among patients with pelvic organ prolapse The highest level of microbial sensitivity was with ciprofloxacin while the least was with cotrimoxazole. It is therefore recommended that ciprofloxacin should be the drug of choice in the treatment of patients with urogenital prolapse especially in the post-operative period.

## Abbreviations

BA: Blood agar
CLED: Cystine lactose electrolyte deficiency
E coli: Escherichia coli
POP: Pelvic organ prolapse
Spp: Specie
TSI: Tripple sugar iron
UTI: Urinary tract infection
VVF: Vesicovaginal fistula

## References

[1] Linda C. Prolapse, 5th edition Dewhurst, Blackwell Science Ltd. London.1996. pg 642–652.

[2] Rabiu KA, Adewunmi AA, Badmus SA, Akinola OI, Akinlusi FM (2009). Pelvic organ prolapse in Lagos. Nigerian Journal of Clinical Medicine.

[3] Okonkwo JEN, Obiechina NJA, Obionu CN (2003). Incidence of pelvic organ prolapse in Nigerian women. J Natl Med Assoc.

[4] TNA J, Jeffcoates TA (2008). Genital Prolapse. Principles of gynaecology. 7th ed.

[5] Lewis AC (1968). Major Gynaecology surgery in the elderly. J Int Fed GynaecolObstet.

[6] Ogunbode O, Aimakhu VE (1973). Uterine prolapse during pregnancy in Ibadan. Am J Obstet Gynaecol.

[7] Cespedes RO, Cross CA, MeGuire EJ (1998). Pelvic prolapse: diagnosing and treating uterine and vaginal vault proplapse. Women’s Health J.

[8] Molton PJD, Studd J (1989). Uterovaginal prolapse. Progress in obstetrics and Gynaecology.

[9] Osinusi BO, Adeleye JA (1976). The symptomatology and clinical presentation of uterovaginal prolapse in Ibadan. Niger Med J.

[10] Tindall VR, Tindall V (1987). Genital Prolapse. Jeffcoates principle of gynaecology.

[11] O’ Leary JA, O’ Laery JL (1990). The extended Manchester operation: a review of 289 cases. Am J Obstet Gynecol.

[12] John CT, Inimgba NM, Ikpeze OC (2009). Uterovaginal prolapse and stress incontinence. Fundamentals of obstetrics and Gynaecology.

[13] Lindsay EN, Sussan B, Richard C, James CR, Anthony S, Thomas MH. Infectious Diseases Society of America guidelines for the diagnosis and treatment of asymptomatic bacteriuria in adults. IDSA guidelines for asymptomatic bacteriuria CID 2005:40 (1 March). 10.1086/427507 15714408

[14] Monga A, Dobbs S. Gynaecology by Ten Teachers. 18th ed. 2006. Chapt 17. Pelvic Organ Prolapse. Bookpower. London. Pg 200–206.

[15] Giacomo NA, Mariangela M, Vincenzo F, Walter A (2006). Critical assessment of pelvic floor surgical reconstruction outcome. European Association of Urologist.

[16] Evans DA, Williams DN, Laughlin LW (1978). Bacteriuria in a population-based cohort of women. J Infect Dis.

[17] Kunin CM, McCormack RC (1968). An epidemiologic study of bacteriuria and blood pressure among nuns and working women. N Engl J Med.

[18] Ojiyi EC, Dike EI, Anolue FC, Nzewuihe AC, Ejikeme CC (2013). Uterovaginal prolapse at a university teaching hospital in south-East Nigeria. Orient Journal of Medicine.

[19] Cheesbrough M. District laboratory practice in tropical countries. Part 2. Cambridge University PressUK. 2000:34–243.

[20] Forbes BA, Sahm DF, Weissfeld AS (2007). Bailey andScott’s diagnostic microbiology.

[21] Okeke TC, Ani VC, Ezenyeaku CCT, Ikeako LC, Enwereji JO, Ekwuazi K (2013). An audit of Uterovaginal prolapse in Enugu, Southeast Nigeria. American. Journal of Clinical Medicine Research.

[22] Rodhe N, Mölstad S, Englund L, Svärdsudd K (2006). Asymptomatic bacteriuria in a population of elderly residents living in a community setting: prevalence, characteristics and associated factors. Fam Pract.

[23] John CT, Okonofua FE, Odunsi K (2003). Genital Prolapse. Contemporary obstetrics and Gynaecology for developing countries.

[24] Ade-Ojo IP, Oluyege AO, Adegun PT, Akintayo AA, Aduloju OP, Olofinbiyi BA (2013). Prevalence and antimicrobial suseptibility of asymptomatic significant bacteriuria among new antenatal enrollees in Southwest Nigeria. International Research Journal of Microbiology (IRJM).

[25] Agersew A, Felek M, Yitayal S, KetemaT, Afework K, Belay A, Abebe A. Bacterial profile and drug susceptibility pattern of urinary tract infection in pregnant women at university of Gondar teaching hospital, Northwest Ethiopia. BMC Research Notes 2012;5:197 http://www.biomedcentral.com/1756-0500/5/197.accessed 2nd August 2014 at 19:15. 10.1186/1756-0500-5-197 PMC3473254 22534117

[26] Aziz MK, Habib-UK, Ihsan-Ullah M, Bushra A, Syed HS (2006). Antimicrobial sensitivity pattern of urine isolates from asymptomatic bacteriuria during pregnancy. Biomedica.

[27] Schnarr J, Smaill F (2008). Asymptomatic bacteriuria and symptomatic urinary tract infections in pregnancy. Eur J Clin Investig.

[28] Tugrul S, Oral O, Kumru P, Köse D, Alkan A, Yildirim G (2005). Evaluation and importance of asymptomatic bacteriuria in pregnancy. Clin Exp Obstet Gynecol.

[29] Rogers RG, Kammerer-Doak D, Olsen A, Thompson PK, Mark D, Walters ES, Lukacz ES, Clifford Q (2004). A randomized, double-blind, placebo-controlled comparison of the effect of nitrofurantoin monohydrate macrocrystals on the development of urinary tract infections after surgery for pelvic organ prolapse and/or stress urinary incontinence with suprapubic catheterization. Am J of Obstetrics and Gynecology.

[30] Adeoye SI, Oladeinde O, Uneke J, Adeoye J (2011). An assessment of asymptomatic bacteriuria among women with VesicoVaginal fistula in south-eastern Nigeria. Nepal Journal of Epidemiology.

[31] Mikael B, Jodal V, William A, Mikael H, Staffan M, Sten R, Michael S, Wettergren B, Susanne J, Catharina S (1996). Interleukin (IL)-6 and IL-8 in children with febrile urinary tract infection and asymptomatic bacteriuria. The journal of lnfectious. Diseases.

[32] Andabati G, Byamugisha J (2010). Microbial aetiology and sensitivity of asymptomatic bacteriuria among ante-natal mothers in Mulago hospital, Uganda. Afr Health Sci.

[33] Ad'hiah AH, Al-Sheikh SKF, Jasim AN, Mayouf KZ (2013). AssessmentOf urinary tract infection and cytokine (IL-2, IL-4 and IL-17A) serum levels in Iraqi samples of systemic autoimmune diseases (rheumatoid arthritis, ankylosing spondylitis and systemic lupus erythematosus) patients. The International Medical Journal.

